# Enhancing Team Identity and Morale With Visual Cues: A Quality Improvement Intervention Using Staff Photo Posters

**DOI:** 10.7759/cureus.91497

**Published:** 2025-09-02

**Authors:** Jamie Sutcliffe, Shahil Rajcoomar, Syed S Ahmed, Obinna J Nzeako

**Affiliations:** 1 Department of Trauma and Orthopaedic Surgery, King's College London, London, GBR; 2 Department of Trauma and Orthopaedic Surgery, Kingston and Richmond NHS Foundation Trust, London, GBR; 3 Department of Trauma and Orthopaedic Surgery, Maidstone and Tunbridge Wells NHS Trust, Royal Tunbridge Wells, GBR

**Keywords:** cohesion, human factor, human factors, photo, quality improvement project, team-building, visual methods, working with a team

## Abstract

Background

Team cohesion and psychological safety are essential for effective clinical care, yet in large or rotating orthopaedic departments, staff may struggle to recall colleagues’ names or feel fully included, especially junior doctors. This is compounded by the shift to more remote and online interaction in the post-COVID world. We introduced a low-cost visual cue intervention to improve team identity, communication, and morale.

Intervention

Photo posters displaying the names and faces of all doctors in the department were created and displayed in key team areas (offices, meeting rooms, and clinics). The aim was to make it easier to recognise and remember team members, improve team spirit, and help junior doctors feel more included.

Methods

This consultant-supported quality improvement project was conducted in a London orthopaedic department. Following the six-month implementation of the posters, a department-wide anonymous survey was distributed to all 24 doctors. The survey included free-response questions assessing the impact of the posters on name recall, team spirit, and junior doctor inclusion.

Results

All 24 doctors responded (100% response rate); 100% reported noticing the posters; 87.5% (n=21) used them to recall a colleague’s name; and 91.7% (n=22) felt the posters improved team spirit. Among the 15 junior doctors (non-consultants), 86.7% reported that the posters contributed to a heightened sense of inclusion within the team.

Conclusion

This simple, low-cost intervention was well received and had a positive impact on team cohesion, name recognition, and junior doctor inclusion. The success of this intervention suggests potential for wider use in similar clinical settings, particularly those with high staff turnover or large multidisciplinary teams.

## Introduction

Problem

Team cohesion and a sense of inclusion are vital in orthopaedic surgery, where complex, high-risk cases often require seamless collaboration between multiple doctors at different levels of seniority. However, following the COVID-19 pandemic, our department experienced a decline in informal interpersonal connections. Contributing factors included mask-wearing, virtual meetings, staff turnover, and split-site working; all of them made it harder for junior doctors to get to know the wider team, recognize senior staff members, and feel part of the department.

This issue was particularly noticeable among newly rotated junior doctors, who often expressed difficulty remembering names and identifying team members for clinical escalation or support. It also affected team morale more broadly, with anecdotal reports suggesting reduced feelings of inclusion, increased hesitancy around communication, and challenges building rapport in the workplace.

Our project took place in a large London teaching hospital with a high-volume orthopaedic department. The team included around 15 junior doctors (FY1 to registrar level) and nine consultants. The patient population served is diverse and includes both elective and acute trauma cases, making strong multidisciplinary coordination essential. We recognized the need for a simple, low-cost, and easily implemented solution to foster team recognition and inclusion, particularly for junior doctors rotating into a new hospital environment.

The project was developed after several informal conversations highlighted this issue, and early feedback suggested that the lack of face-to-face familiarity contributed to avoidable friction in communication and team functioning. We therefore designed a visual cue intervention in the form of a laminated photo poster showing all junior doctors’ names, grades, and headshots, placed in key areas throughout the department.

Background

Teamwork and a sense of belonging are critical to delivering safe, high-quality care in surgical specialties, where coordinated action and timely escalation are often essential to patient outcomes [1,2]. In the aftermath of the COVID-19 pandemic, healthcare teams across the National Health Service (NHS), particularly in hospital-based services, which were used to face-to-face interaction, faced new barriers to interpersonal connection. Changes such as increased service demand, mask-wearing, split-site working, and remote meetings disrupted informal socialisation, leading to reduced name recognition, team cohesion, and psychological safety [3-5].

This was especially evident among junior doctors, whose frequent rotations and shorter placements make it harder to build rapport and feel part of a cohesive team. While structured tools such as surgical safety checklists have effectively reduced preventable harm [6], growing evidence highlights that interventions targeting interpersonal dynamics such as communication, trust, and inclusion can also improve staff morale, reduce burnout, and enhance team performance [1,7]. Visual cues such as photographs can enhance name recall, foster psychological safety, and contribute to shared team identity, key elements that support effective teamwork in clinical settings [8].

Rationale

To address these concerns, we introduced a simple visual cue: a photographic poster showing the names, grades, and headshots of all junior doctors in the department. The poster was displayed in high-traffic clinical areas (e.g., clinic areas and handover rooms) for six months. We hypothesised that increasing visual familiarity would help doctors remember each other’s names, improve perceived morale and team spirit, and strengthen the sense of inclusion among junior team members.

Specific, measurable, achievable, relevant, and time-bound (SMART) aim

Our aim was to improve team cohesion and inclusion within the orthopaedic department by increasing name recognition, morale, and junior doctor inclusion through a photographic poster intervention over a six-month period. We set out to achieve >80% agreement among respondents that the poster improved name recall, team spirit, and feelings of inclusion among junior doctors by the end of the project.

## Materials and methods

Context

This quality improvement project was undertaken in a London orthopaedic department composed of 24 doctors spanning all training grades, from foundation doctors to consultants. Post-COVID-19 service changes, including remote handovers, shift-based working, and prolonged mask usage, had reduced informal team bonding opportunities, contributing to diminished team cohesion and name recognition.

Intervention

The intervention consisted of a laminated A3 poster displaying the names, training grades, and headshots of all junior doctors in the department. The aim was to support interpersonal familiarity, foster inclusion, and strengthen team identity. Posters were deliberately placed in high-traffic shared spaces, including the doctors’ office, meeting rooms, and fracture clinics, to maximise visibility and repeated exposure. The intervention was maintained in place for six months. The posters were put up by one of the registrars involved in the research team.

Study of the intervention

Following the intervention period, a survey was distributed to assess its perceived impact. The survey consisted of five items and was delivered electronically. It targeted the full cohort of 24 doctors, gathering both quantitative and qualitative data to evaluate changes in team dynamics. The survey included Likert-style and multiple-choice questions alongside open-ended text boxes for qualitative feedback.

Measures

The outcomes of interest were as follows: (1) Name recall: whether the poster helped doctors remember the names of colleagues; (2) Team spirit: whether the intervention improved perceived morale within the team; (3) Inclusion: especially among junior doctors, whether the intervention improved their sense of being part of the team. These outcomes were selected based on the goals of improving team dynamics, psychological safety, and identity within a large, rotating clinical team.

Analysis

Quantitative survey results were summarised using descriptive statistics (e.g., percentages of affirmative responses). Thematic analysis was applied to qualitative feedback using an inductive approach. Two researchers reviewed the open-text responses to identify common themes related to visibility, approachability, and psychological safety. The manuscript was then composed according to the Standards for the Quality Improvement Reporting Excellence 2.0 (SQUIRE 2.0) reporting guidelines [9].

Ethical considerations

This project was registered as a quality improvement initiative and did not require formal research ethics approval. All survey responses were anonymous, and participation was voluntary. No patient data were used.

## Results

Participant characteristics

A total of 24 orthopaedic doctors from a London teaching hospital participated in the survey, comprising nine consultants, seven registrars, six senior house officers, and two foundation year one doctors. The response rate was 100%, with no missing data.

Intervention exposure and engagement

The intervention, a laminated poster displaying the names, roles, and headshots of junior doctors, was implemented for six months without modification. It was displayed in high-traffic areas of the department, including offices, meeting rooms, and fracture clinics, to maximise visibility. The included questions can be found in the Appendix (Table 1).

**Figure 1:**
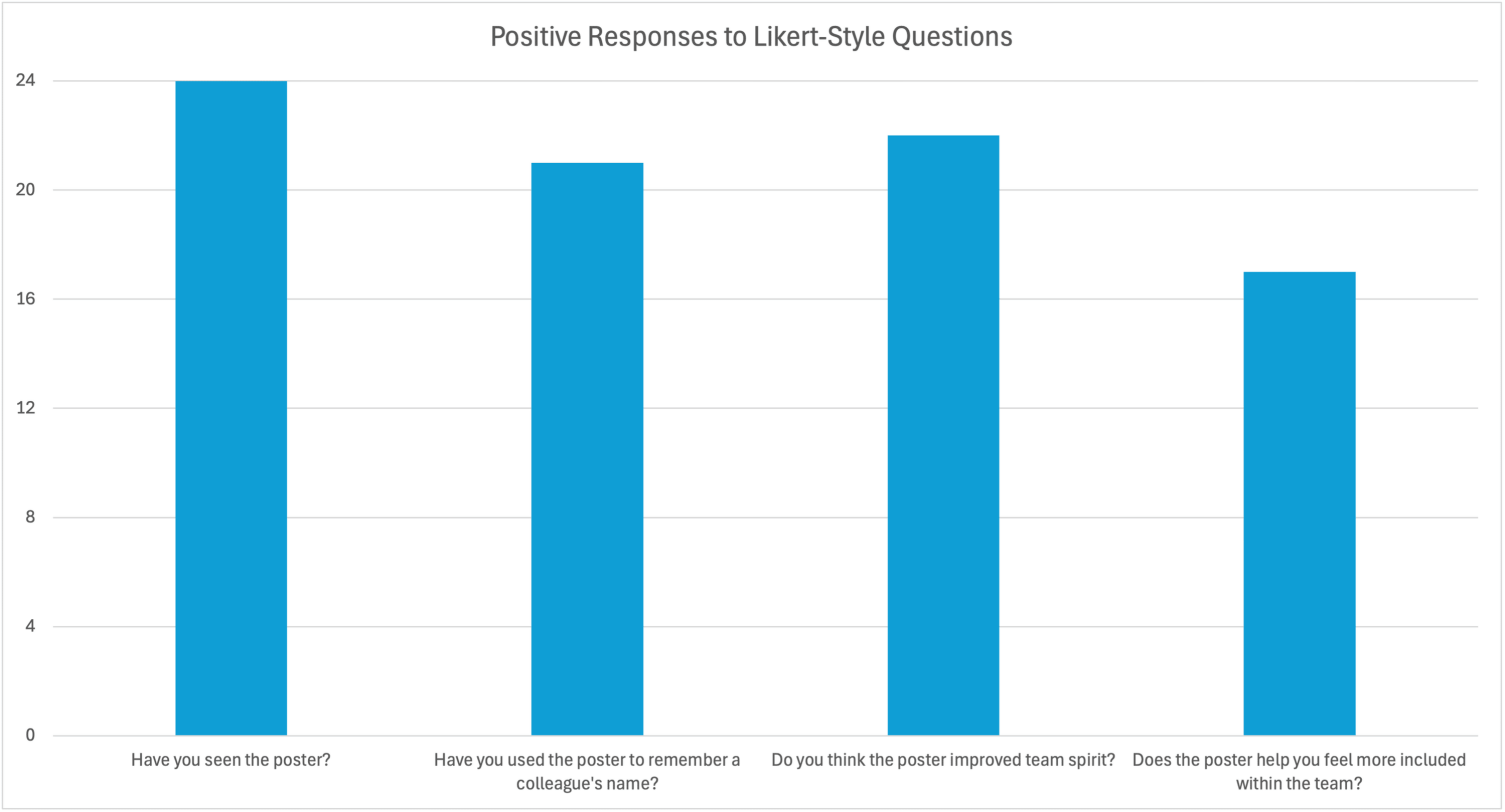
Graph showing the number of positive responses to the four-point Likert-style questions in the questionnaire out of the total (n=24) number of responses

All 24 respondents reported having seen the poster, and 21 (87.5%) reported using it to help recall a colleague’s name. The majority (22 respondents, 91.7%) felt the poster improved overall team spirit. Among junior doctors (foundation year to registrar level), 13 of 15 respondents (86.7%) reported that the poster helped them feel more included in the team. See Figure 1 for the results.

Associations and perceived impact

Qualitative responses supported the quantitative findings. Several junior doctors noted that the poster made it easier to identify senior staff, improving their confidence in clinical escalation and enhancing their sense of team integration. Consultants who responded to the open-text section recommended extending the poster to include all grades of staff, indicating a desire for broader inclusion.

Contextual influences

This project was implemented in the context of ongoing post-pandemic workforce disruptions, including increased use of face masks, remote meetings, and frequent staff turnover. These contextual factors may have amplified the perceived value of the intervention by increasing the barriers to interpersonal connection.

Unintended consequences

No negative consequences were reported. Four consultants suggested expanding the poster to include consultants, emphasizing the importance of representing all team members. Several junior doctors highlighted the poster's usefulness in identifying senior doctors for the escalation of clinical concerns, demonstrating its practical value in a clinical context.

## Discussion

Summary

This project found that a simple, low-cost poster featuring junior doctors’ names, grades, and photographs significantly contributed to improved team cohesion in an orthopaedic department. The majority of respondents reported using the poster to recall colleagues’ names, noted improvements in team spirit, and, among juniors, reported feeling more included within the team. These outcomes align with the original aims of the project and suggest that visual familiarity can positively influence workplace culture. A particular strength of the intervention was its high acceptability, as demonstrated by 100% exposure and survey response rate.

Interpretation

The findings suggest a strong association between the intervention and perceived improvements in communication, morale, and inclusion. The poster likely served as a prompt for interpersonal connection, especially in a clinical context where masks, shift work, and physical separation reduce organic social interactions. These results echo themes in existing literature on psychological safety, team identity, and communication in healthcare, although few studies have evaluated such visual interventions in this specific context [1-3].

The impact of the poster extended beyond social cohesion. Several junior doctors commented that it facilitated clinical escalation by helping them identify senior staff, indicating a possible secondary benefit in patient safety. Contextual factors, such as staff turnover and pandemic-related barriers to in-person interaction, may have amplified the intervention’s perceived value. The primary unanticipated outcome was strong interest from consultants in expanding the poster to include senior team members.

This intervention required minimal resources to implement, did not interfere with other departments, and could rapidly be rolled out in other departments and in other hospitals, too.

Limitations

This was a single-centre project without a control group, limiting generalisability. Self-reported survey responses introduce potential bias, and the anonymous format prevented correlation of outcomes with individual characteristics (e.g., job grade or time in post). No objective clinical outcomes (e.g., incident reports or patient safety markers) were assessed. Nevertheless, efforts to minimise bias included ensuring anonymity and encouraging both closed and open responses for richer feedback.

## Conclusions

This work supports the utility of visual team-building interventions in clinical environments, particularly where traditional social cohesion is challenged. The intervention was well-received, low-cost, and potentially sustainable over time. It could be easily adapted for other departments or specialties, especially if expanded to include the entire multidisciplinary team. Further study could include a pre-/post-design with control groups or expansion across multiple sites. Next steps may involve integrating such posters into onboarding processes or evaluating their impact on patient safety indicators and further assessing their impact on junior doctor wellbeing.

## Appendices

**Table 1:** Questions included in the questionnaire

No.	Question	Answer Type
1	Have you seen the poster?	Likert Scale
2	Have you used the poster to remember a colleague's name?	Likert Scale
3	Do you think the poster improved team spirit?	Likert Scale
4	Does the poster help you feel more included within the team?	Likert Scale
5	Any other comments?	Free Response
